# Portability of a Small-Molecule Binding Site between Disordered Proteins

**DOI:** 10.3390/biom12121887

**Published:** 2022-12-16

**Authors:** Rajesh Jaiprashad, Sachith Roch De Silva, Lisette M. Fred Lucena, Ella Meyer, Steven J. Metallo

**Affiliations:** 1 Department of Chemistry, Georgetown University, Washington, DC 20057, USA; 2 Institute for Soft Matter Synthesis and Metrology (ISMSM), Georgetown University, Washington, DC 20057, USA

**Keywords:** intrinsically disordered proteins, Myc, protein-protein interaction, drug targets, SLiM, small-molecule inhibitors

## Abstract

Intrinsically disordered proteins (IDPs) are important in both normal and disease states. Small molecules can be targeted to disordered regions, but we currently have only a limited understanding of the nature of small-molecule binding sites in IDPs. Here, we show that a minimal small-molecule binding sequence of eight contiguous residues derived from the Myc protein can be ported into a different disordered protein and recapitulate small-molecule binding activity in the new context. We also find that the residue immediately flanking the binding site can have opposing effects on small-molecule binding in the different disordered protein contexts. The results demonstrate that small-molecule binding sites can act modularly and are portable between disordered protein contexts but that residues outside of the minimal binding site can modulate binding affinity.

## 1. Introduction

Proteins exist along a conformational spectrum from fully folded and well-structured proteins to unstructured or intrinsically disordered proteins (IDPs) [1,2]. Many proteins lie between these two endpoints and contain both ordered regions as well as substantial (>40 amino acids) intrinsically disordered regions (IDRs) [3,4]. While structured regions fluctuate around a clear average conformation, IDPs and IDRs exist as a rapidly fluctuating series of conformations [5]. An IDR can be described as an ensemble of conformations with low energy barriers for interconversion [6]. Protein disorder is found throughout biological systems and is particularly prevalent in complex eukaryotes [7]. Within cells, IDPs and IDRs perform many crucial functions and are particularly prevalent in signal transduction and transcriptional control with greater than 80% of transcription factors predicted to be partially or completely disordered [8,9].

Proteins containing disordered regions are also overrepresented in pathological conditions such as cancer and neurodegenerative diseases [10,11]. A contributing reason for a central role of IDRs in both normal cellular functions and in pathologies is the ability of IDRs to act as sites of molecular recognition [12,13,14]. Within cells, the formation of many biomolecular condensates has been shown to be driven by molecular recognition functions of disordered proteins [15]. Through dynamic and multivalent interactions with other proteins or with nucleic acids, typically RNA, IDRs are able to mediate the formation and properties of many of the biomolecular condensates in cells [16,17]. These membraneless organelles function in crucial processes such as RNA splicing, modulation of reaction rates, and transcription control, among others [18].

While many IDRs participate in highly dynamic interactions, IDRs can participate in protein–protein interactions (PPIs) with a range of affinities and kinetic stabilities [19]. Interactions also occur with a range of disorder present in the complex. Certain IDRs undergo coupled folding and binding in the formation of a complex [20]. Some IDRs adopt different conformations when bound to different partners [21]. Other IDRs form complexes while remaining disordered [22]. Within larger disordered domains, portions of sequence that mediate protein–protein interactions via coupled folding and binding to structured partners are referred to as molecular recognition features (MoRFs) [13]. MoRFs were recognized as potentially useful starting points in developing inhibitors of PPIs [23] and can be predicted within disordered sequences [24,25]. Post-translational modifications (PTM) often involve recognition of a disordered modification site. Of the characterized phosphorylation sites, 84% percent are in disordered regions [26]. These PTM sites are an example of short (3–10 residues) recognition sequences that are found in disordered regions and that can mediate specific domain interactions. These short sequences, which overlap with MoRFs, are called short linear motifs, SLiMs [27]. In both MoRFs and SLiMs, the disordered nature of the target is important in allowing access to its binding partner. The sequences are not sequestered in a folded context and therefore are available for binding with access to the chemical moieties along the entire sequence [28].

In addition to mediating interactions between biomolecules, disordered regions were also found to support binding by small molecules. Early studies involved targeting of the disordered, monomeric bHLHZip region of the c-Myc oncoprotein (Myc) with the goal of interfering with the coupled folding and binding of Myc to its obligate heterodimerization partner Max [29,30]. Myc is dysregulated in a majority of human cancers [31] and even transient inhibition of Myc activity can cause cancer cells to differentiate [32]. Consequently, Myc activity has been targeted in a wide array of mechanisms [33,34,35]. The crucial biological function of Myc drove the direct targeting of Myc, in spite of its disordered character, and caused it to become an early test case for the direct targeting of disordered proteins with small molecules [36]. Subsequently, a range of disordered proteins with a variety of functions have been demonstrated as targets of small molecules with a concentration on transcription factors and neurological disease-related targets [37,38,39,40]. Despite progress, with an increasing scope of small-molecule IDP interactions reported, we still do not have a clear understanding of the major factors controlling what constitutes a disordered sequence that supports small-molecule affinity, nor do we know how binding site specificity is achieved in these interactions that appear to remain dynamic and exposed to solvent in the complex.

In order to better understand the binding of small molecules to disordered sequences, we sought to investigate potential parallels between small-molecule IDR interactions and SLiM interactions with partner proteins. Both SLiMs and disordered small-molecule binding sites consist of short linear sequences that mediate specific binding with an interaction partner, either a protein partner or a molecular partner [27,36]. We sought to determine if disordered small-molecule binding sites could recapitulate the ability of SLiM sequences to recognize their specific binding partner in a modular fashion, using the same (or similar) recognition sequence embedded in different protein contexts to bind to the same partner [41]. Here, we ported a specific small-molecule recognition sequence between two disordered proteins and demonstrated that the small-molecule binding function moved along with the sequence. Further, we found that residues flanking the binding site modulated binding affinity as in other IDR recognition motifs.

## 2. Materials and Methods

### 2.1. Myc_353–437_, MaxRH, Max, and Myc_402–412_ Purification

The coding sequences for Myc_353–437_, MaxRH, P21 Max, and P22 Max were designed to include a hexahistidine (6xHis) tag, and a tobacco etch virus (TEV) recognition site immediately prior to the protein coding region (Figure S1). The Myc_353–437_ coding sequence was inserted into a pET23d+ plasmid (Genscript) while MaxRH was inserted into a pET24d+ plasmid (Genscript). Max isoforms (P21 and P22) were expressed from previously described pET151D-TOPO plasmids [42]. The Myc_353–437_ A401E, E410N, and MaxRH-N78E mutants were generated using QuickChange Lightning Mutagenesis (Agilent) following the manufacturer’s protocol. MaxRH-Y115F/Y123F mutagenesis was conducted on the MaxRH plasmid by Genscript.

The 6xHis-tagged proteins were expressed in BL21(DE3) pLysS *E. coli* cells (Invitrogen) under autoinducing conditions following a protocol by Studier [43]. The cells were grown in a medium containing 1% *w*/*v* N-Z amine, 0.5% *w*/*v* yeast extract, 25 mM Na_2_HPO_4_, 25 mM KH_2_PO_4_, 50 mM NH_4_Cl, 5 mM Na_2_SO_4_, 2 mM MgSO_4_, 0.5 % *v*/*v* glycerol, 0.05 % *w*/*v* glucose, 0.2 % *w*/*v* lactose and a trace-metals mix of 10 µM FeCl_3_, 4 µM CaCl_2_, 2 µM MnCl_2_, 2 µM ZnSO_4_, 0.4 µM CoCl_2_, 0.4 µM CuCl_2_, and 0.4 µM NiCl_2_. A single colony of the bacterial culture was grown for 18 h at 37 °C in a shaking incubator at 200 rpm. Cells were collected by centrifugation at 9000 rpm for 30 min using a Sorvall RC 6+ centrifuge (Thermo Scientific, Marietta, OH, USA). The supernatant was discarded, and cells were lysed by sonication in 50 mL of lysis buffer containing 8 M urea, 100 mM Tris-HCl, and 10 mM sodium phosphate at pH 8.0. Cell debris was removed by centrifugation at 18,000 rpm for 30 min. The lysate was loaded onto a nickel nitriloacetate (Ni-NTA) affinity resin (GoldBio) column to purify the proteins using a pH gradient, where the column was equilibrated with lysis buffer at pH 8, and non-specific proteins were removed using a wash buffer at pH 6.4 (8 M urea, 100 mM Tris-HCl, and 10 mM sodium phosphate). An elution buffer at pH 4.5 (8 M urea, 100 mM Tris-HCl, and 10 mM sodium phosphate) was then used to elute 6xHis-tagged proteins bound to the Ni-NTA column. The elutions were pooled and buffer exchanged into 50 mM MES at pH 5 using 3000 MW cutoff Amicon ultrafiltration units (Millipore-Sigma, Billerica, MA, USA). After buffer exchanging into MES, the protein was incubated overnight with a TEV protease to cleave the 6xHis-tag. His-tag cleavage was confirmed using SDS-PAGE. Once cleavage was confirmed, the TEV protease activity was quenched by adding urea, and the protein was then buffer exchanged into 8M urea lysis buffer at pH 8. A second Ni-NTA column was used to remove uncleaved protein, 6xHis-tag, and 6xHis-tagged TEV. The cleaved protein was dialyzed against HPLC grade water containing 0.1% trifluoroacetic acid (TFA) using a 3000 MW cutoff dialysis membrane. All proteins were further purified through reverse phase HPLC (Vydac-C18) with a water/acetonitrile gradient containing 0.1% TFA and purified to >95% purity. The proteins were lyophilized and stored at −20 °C. The Myc_402–412_ peptide, Ac-YILSVQAEEQK-NH_2_, was synthesized by Genscript using solid phase peptide synthesis. The peptide was reconstituted in HPLC grade water and further purified through reverse phase HPLC (Vydac-C18) with a water/acetonitrile gradient and 0.1% TFA (Sigma-Aldrich, Saint Louis, MI, USA). The peptide was lyophilized and stored at −20 °C.

### 2.2. Preparation, Characterization and pKa Determination of 34RH

The small molecule (Z)-4-((4-oxo-2-thioxothiazolidin-5-ylidene)methyl)benzoic acid (hereafter referred to as 34RH) was previously synthesized according to established procedures, and the structure was confirmed by ^1^H and ^13^C NMR (Figure S6) using a 400 MHz spectrometer [44]. The dry compound was stored at 4 °C. Stock solutions (1 mM) of 34RH were made using either dimethyl sulfoxide (DMSO) or ethanol and stored at −20 °C. Fluorescence and dynamic light scattering (DLS) experiments were performed using DMSO, while circular dichroism (CD) was conducted using ethanol to avoid the high absorbance of DMSO at short wavelengths. DLS measurements were performed on 34RH using an LS Spectrophotometer (LS Instruments) at 25 °C (Figure S3). The dispersant viscosity was set for water. Samples were analyzed in 1xPBS (pH 7.4) and 5% total DMSO. The compound was serially diluted two-fold from 100 μM to a final concentration of 6.25 μM. Samples were placed into 5 mm cylindrical glass cuvettes (LS Instruments) and measured using a 600 nm laser at a 90° angle for 20 s. The data were analyzed using LS Spectrophotometer software provided by the manufacturer.

The imide pKa of 34RH was determined using UV/Vis by measuring the absorbance of 10 µM of 34RH in 1xPBS at various pH values using an Agilent 8453 UV/Vis spectrophotometer (Figure S2). The absorbance at 327 nm for each pH value was fit to a curve using the Henderson-Hasselbalch (shown in Equations (1) and (2)), where c is the pKa, x is the pH, a is the signal for the fully protonated acid, and b is the signal for the fully ionized base [45].

(1) $$\mathrm{pKa}-\mathrm{pH}=\log\;\frac{\left[\mathrm{acid}\right]}{\left[\mathrm{base}\right]}\;$$

(2) $$y=\frac{a+b*10^{\left(c-x\right)}}{1+10^{\left(c-x\right)}}$$

### 2.3. Tyrosine Fluorescence Quenching Assay

The lyophilized protein (or peptide) was reconstituted in ultrapure water and incubated to room temperature for at least 1 hr. The protein or peptide was then sterile filtered using a pre-wet 0.2 μm polyethylene sulfone (PES) filter (VWR), and the concentration was determined by the absorbance at 274 nm using the extinction coefficient per tyrosine of ε_274_ = 1470 M^−1^cm^−1^. The final stock concentrations ranged from 50 to 100 μM.

For excess 34RH tyrosine fluorescence quenching experiments, samples were prepared with the following buffer components: sterile filtered water, 1xPBS (137 mM NaCl, 2.7 mM KCl, 4.3 mM Na_2_HPO_4_, 1.4 mM KH_2_PO_4_), and 5% total DMSO. The pH of the buffer was adjusted to 6 or 7.4 depending on the experiment performed. Three separate samples were prepared in 1xPBS and 5% total DMSO: one containing 50 μM 34RH alone, one containing 1 μM of protein and 50 μM 34RH, and one containing 1 μM protein alone. For the 34RH containing samples, the compound was delivered from a 1 mM DMSO stock. These samples were then serially diluted two-fold from 50 μM 34RH to 1.56 μM 34RH. All samples for fluorescence measurements maintained a final 5% DMSO concentration.

The samples were incubated for at least 15 min before fluorescence was measured. The samples were excited at 274 nm, and the emission spectra were obtained from 285 to 340 nm using 5 nm excitation and 5 nm emission slits using a Horiba Fluoromax 4 fluorometer. The fluorescence data were background corrected using buffer (for samples with only protein) or small molecule (for samples containing protein and 34RH) (Figure S4). The absorbance due to 34RH and protein at the excitation and emission wavelengths can suppress the observed fluorescence intensity to give rise to the inner filter effect [46]. To account for this suppression, we corrected the fluorescence signals using Equation (3). This correction accounted for the fluorescence suppression due to the absorbance of both 34RH and the protein [47].

(3) $$F_{\mathrm{corr}}=F_{\mathrm{obs}}*\;10^{\frac{\left(A_{\mathrm{ex}}+{\;A}_{\mathrm{em}}\right)}{2}}$$

Here, the corrected fluorescence is F_corr_, the background-subtracted observed fluorescence is F_obs_, and A_ex_ and A_em_ are the total absorbance at the excitation and emission wavelengths, respectively. The amount of protein fluorescence quenched by 34RH at a particular concentration was calculated using F_corr_ at λ = 304 nm (Equation (4)).

(4) $$\mathrm{Fraction}\;\mathrm{Quenched}=1-\frac{{\mathrm{Fcorr}}_{304\mathrm{nm}}\;\mathrm{of}\;\left(\mathrm{Protein}\;+34\mathrm{RH}\right)}{{\;\mathrm{Fcorr}}_{304\mathrm{nm}}\;\mathrm{of}\;\mathrm{Protein}\;\mathrm{only}}$$

The quenching data were fit to a Langmuir binding isotherm using Equation (5), from which the dissociation constant (K_D_) was obtained [48]. Here, Qmax describes the maximum fraction quenched, [L]_T_ is the total 34RH concentration, and [P]_T_ is the total protein concentration. The total concentration of the protein was 1 µM for all fluoresce experiments conducted at a constant protein concentration.

(5) $$\mathrm{Fraction}\;\mathrm{Quenched}=\mathrm{Qmax}*\left[\frac{({\left[L\right]}_{T}+\left[P{]}_{T}+{\;K}_{D}\right)-\sqrt{(\left([L{]}_{T}+[P{]}_{T}+{\;K}_{D}{)}^{2}-4\;*\;[L{]}_{T}\;*\;[P{]}_{T}\right)}}{2*{\left[L\right]}_{T}}\right]$$

### 2.4. Circular Dichroism (CD)

Samples containing Myc_353–437_, Myc_402–412_ peptide, MaxRH, Max isoforms, or mutants in the absence and presence of excess concentrations of 34RH were prepared in 1xCD buffer (50 mM KF, 4.3 mM NaH_2_PO_4_, 1.4 mM KH_2_PO_4_, 5% ethanol). The pH of the buffer was adjusted to either 6 or 7.4 depending on the experiment conducted. The compound was delivered from a 1 mM ethanol stock solution. Samples were incubated for 1 hr before measurement. The far UV-spectra of the proteins and peptide were recorded in a quartz cuvette with a path length of 0.1 cm using a Jasco J720 spectropolarimeter. The samples were scanned from 270 to 195 nm with an increment of 1 nm, constant bandwidth of 10 nm, and a scanning speed of 1 nm per minute. After subtracting the buffer signal, the raw data in millidegrees was converted to mean residue ellipticity (MRE).

## 3. Results

### 3.1. Binding of the Small Molecule 34RH to the Myc Target Site

**Figure 1:**
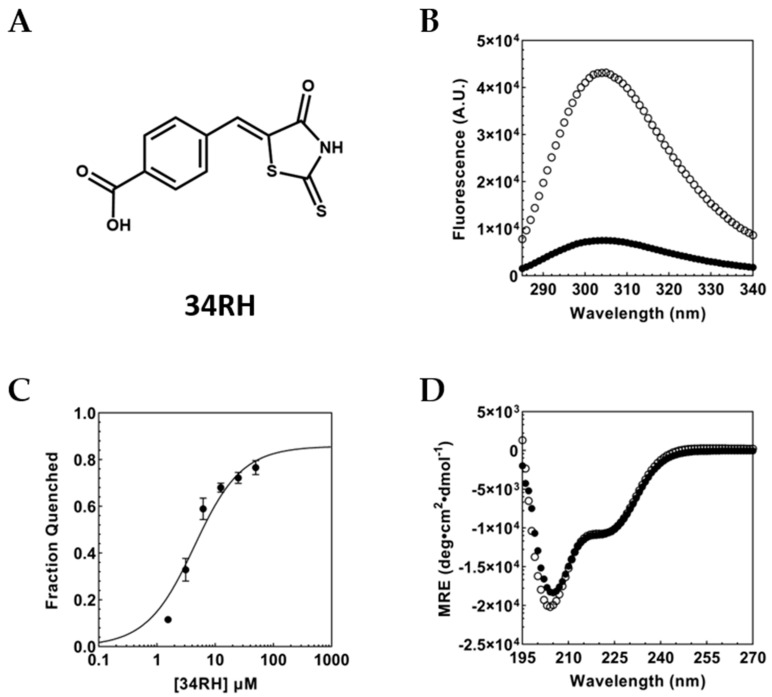
(**A**) Structure of 34RH. (**B**) Inner filter corrected fluorescence emission spectrum of 1 μM Myc_353–437_ with (black circles) and without (white circles) 50 μM 34RH in 1xPBS at 25 °C, pH 7.4. (**C**) Equilibrium titration of 1 μM Myc_353–437_ with excess 34RH fit to a Langmuir binding isotherm, K_D_ = 3.9 ± 1.3 µM. Error bars represent the standard error of three independent trials. (**D**) Circular dichroism of 2.5 µM Myc_353–437_ with (black circles) and without (white circles) 50 µM 34RH in 1xCD buffer.

Previously, Yin and coworkers demonstrated that the small molecule 10058-F4 (1RH) disrupted Myc-Max dimerization [30]. Subsequently, we identified the specific interaction site of 1RH within the disordered, monomeric Myc bHLHZip domain [49]. In this study, we use the previously reported 1RH-derivative, 34RH—which maintains the core structure of 1RH, while replacing an ethyl group with a carboxylic acid moiety on the phenyl ring (Figure 1A) [44]. At neutral pH, 34RH is present primarily in the dianionic form as the pKa of the imide group of the rhodanine heterocycle is 5.3 ± 0.3 (Figure S2) and shows good solubility based on dynamic light scattering (Figure S3).

The binding site of 1RH in Myc_353–437_ had been previously localized to within residues 402 to 412 [49]. In Myc_353–437_, the only fluorescent residue (Tyr or Trp) is Tyr_402_ located in the binding site. We and others have demonstrated that the interaction with 1RH causes quenching of Tyr_402_ [49,50]. Here, we exploited this tyrosine fluorescence to evaluate binding of the 34RH molecule to Myc_353–437_. Upon addition of 34RH to Myc_353–437_, we observed that the Myc_353–437_ fluorescence was quenched (Figure 1B).

The observed fluorescence quenching was titratable, and the 34RH binding affinity to Myc_353–437_ was determined by monitoring tyrosine fluorescence as a function of 34RH concentration. The quenching data was fit to a Langmuir binding isotherm yielding a dissociation constant (K_D_) of 3.9 ± 1.3 µM (Figure 1C). Notably, the dissociation constant obtained for 34RH and Myc_353–437_ is comparable to the previously determined K_D_ for 1RH and Myc_353–437_ of 5.3 ± 0.7 µM [49]. In addition to titrations with 34RH in excess over Myc_353–437_, we performed titrations with equimolar concentrations of Myc_353–437_ and 34RH, where we observed that the Myc_353–437_ fluorescence was quenched to a comparable extent, and we obtained a similar K_D_ of 5.9 ± 0.8 µM (Figure S5).

We performed circular dichroism (CD) experiments with and without 34RH to determine if the addition of 34RH altered the average conformation of Myc_353–437_. The CD spectrum of Myc_353–437_ indicated that the domain was largely disordered with some α-helical character, as expected from NMR experiments on Myc [51,52,53]. Those NMR experiments indicated that the Myc sequence was predominantly random coil but with partial helical character, particularly in the region around residues 360–370 and with strong helical character from residues 416–422. Comparison of the CD spectra of Myc_353–437_ with and without the addition of the small molecule indicated that 34RH did not substantially alter the average conformation of the protein (Figure 1D).

### 3.2. Binding of 34RH to the Myc_402–412_ Peptide

**Figure 2:**
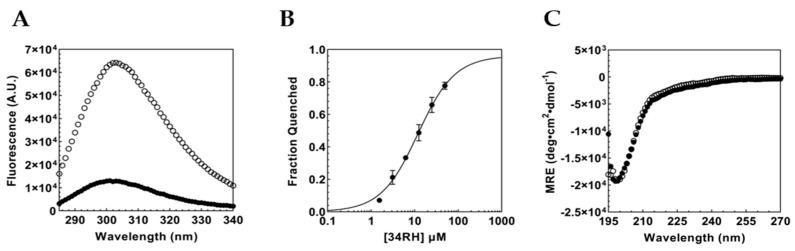
(**A**) Inner filter corrected fluorescence emission spectrum of 1 µM Myc_402–412_ peptide with (black circles) and without (white circles) 50 µM 34RH. (**B**) Fraction quenched titration curve for 1 µM Myc_402–412_ peptide and 34RH fitted to a Langmuir binding isotherm yielding a K_D_ = 11.5 ± 1.2 µM. Error bars represent the standard error of three independent trials. (**C**) CD spectrum of 2.5 µM Myc_402–412_ peptide with (black circles) and without (white circles) 50 µM 34RH.

Our previous studies showed that small molecules can bind to short contiguous segments in Myc_353–437_ [42]. Guided by mutations and truncations, we demonstrated that the small molecule 1RH could bind to the short peptide sequence Myc_402–412_ [49]. Here, we used this peptide, Y_402_ILSVQAEEQK_412_, to determine the affinity of 34RH for the isolated binding site. As with Myc_353–437_, binding of 34RH to the peptide was monitored via Tyr fluorescence quenching (Figure 2A). In the context of the peptide, we again observed strong fluorescence quenching and titratable binding. From the data, we obtained a K_D_ of 11.5 ± 1.2 µM, within three-fold of the affinity determined for Myc_353–437_. The dissociation constant for the isolated peptide sequence is similar to the previously reported binding affinities of 1RH for the Myc_402–412_ peptide of between 13 and 14 µM [49,50].

To monitor the conformation of the peptide upon introducing 34RH, we performed CD. We observed that the peptide displayed a single negative MRE at 202 nm, indicating a predominantly random-coil conformation. Upon addition of 34RH, the peptide does not exhibit perturbations to the structural ensemble, as observed by the near identical CD spectra with and without the compound. The result with 34RH contrasts with that of the previous data with 1RH, where the addition of 1RH induced a substantial shift in the peptide’s secondary structure [49]. The lower concentration of the peptide (2.5 µM versus 20 µM) and the charged nature of 34RH potentially account for the differences in the structural perturbation. Our results illustrate that the small molecule 34RH can bind to a short segment of Myc_353–437_ independent of the entire protein domain and without imparting significant structural alterations. Furthermore, 34RH can bind to the random coil, indicating that a disordered eleven-residue peptide is sufficient for the binding of the small molecule.

### 3.3. Portability of the Small-Molecule IDP Binding Site

**Figure 3:**
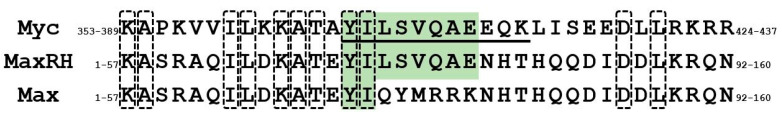
Alignment of the binding site region of Myc_353–437_ with MaxRH and Max. Outlined residues are identical. Underlined residues denote the Myc_402–412_ sequence. Highlighted green residues represent the overlap of the minimal binding site between the proteins.

Short linear motifs (SLiMs) or eukaryotic linear motifs (ELMs) use the same or closely related sequences to bind partner proteins in different contexts [27]. The short linear binding site of 34RH is similar to a SliM since binding occurs independently of the larger context while maintaining affinity. If 34RH binding to the peptide sequence is truly independent of the overall context, we should be able to move the binding sequence into a different protein and recapitulate 34RH binding activity in that new context. Here, we chose Max, a heterodimerization partner of Myc [54] previously shown not to interact with 1RH [30], to receive the ported binding sequence. The canonical isoform of Max (P22 Max) is 160 amino acids in length and shares a 38% sequence identity with Myc in the bHLHZip region. Max has a short N-terminal disordered region and a longer disordered C-terminus [55]. We aligned Myc_353–437_ and Max and compared the binding site region (Figure 3). The comparison indicated that the Max sequence, Y_70_IQYMRRK_77_, aligned with the binding site in Myc. Beyond the first two residues of this site, the Max sequence lacks identity with Myc in the binding region. We wanted to mutate a minimal set of amino acids in Max to form the small molecule binding sequence. Previously, we determined that the 370–409 sequence of Myc, but not 353–405, could bind to 1RH [49]. Together with the Myc_402–412_ binding data, we used this information to demarcate the minimal binding site of Y_402_ to E_409_. Therefore, we mutated six residues (Q_73_YMRRK_77_) in Max, in order to match the 402–409 region of Myc_353–437_ (Figure 3). This new construct, termed MaxRH, contained what we postulated to be a minimal, functional 34RH binding sequence (-YILSVQAE-) ported into Max.

**Figure 4:**
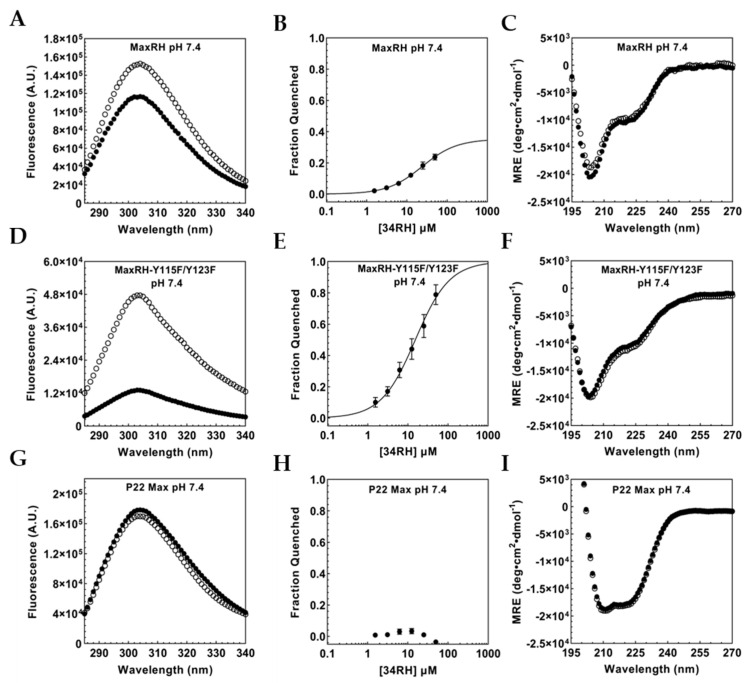
(**A**) Inner filter corrected fluorescence emission spectrum of 1 µM MaxRH with (black circles) and without (white circles) 50 µM 34RH. (**B**) Fraction quenched titration curve for 1 µM MaxRH with 34RH fit to a Langmuir binding isotherm, K_D_ = 23.4 ± 1.1 µM (**C**) CD spectrum of 1 µM MaxRH with (black circles) with without (white circles) 50 µM 34RH. (**D**) Inner filter corrected fluorescence emission spectrum of 1 µM MaxRH-Y115F/Y123F with (black circles) and without (white circles) 50 µM 34RH. (**E**) Fraction quenched titration curve for 1 µM MaxRH-Y115F/Y123F with 34RH fit to a Langmuir binding isotherm, K_D_ = 14.9 ± 1.9 µM (**F**) CD spectrum of 1 µM MaxRH-Y115F/Y123F with (black circles) with without (white circles) 50 µM 34RH. (**G**) Inner filter corrected fluorescence emission spectrum of 1 µM P22 Max with (black circles) and without (white circles) 50 µM 34RH. (**H**) Fraction quenched titration curve for 1 µM P22 Max with 34RH (**I**) CD spectrum of 1 µM P22 Max with (black circles) with without (white circles) 50 µM 34RH at pH 7.4.

As previously described for Myc_353–437_, we monitored 34RH binding to MaxRH via tyrosine fluorescence quenching (Figure 4A). MaxRH contains three tyrosine residues in total, one in the binding sequence (Tyr_70_) and two in the disordered C-terminus (Tyr_115_ and Tyr_123_). We expect Tyr_115_ and Tyr_123_ not to quench in the presence of 34RH while Tyr_70_, in the generated binding site, should exhibit titratable quenching. If we successfully ported over the 34RH binding site, we would observe titratable quenching but with a lower maximum fraction quenched (in comparison to Myc_353–437_) due to Y_115_ and Y_123_ retaining their fluorescence. In the presence of 50 µM 34RH, MaxRH tyrosine fluorescence is quenched (Figure 4A). Titration of a constant concentration of MaxRH with 34RH yielded a binding curve with a dissociation constant of 23.4 ± 1.1 µM (Figure 4B) and the expected lower maximum quenching. The K_D_ for MaxRH:34RH indicates that 34RH can bind to the ported sequence in a new context, albeit with reduced affinity. We also tested MaxRH with and without 50 µM of 34RH using CD (Figure 4C). The CD of MaxRH in the absence of compound showed a spectrum similar to Myc_353–437_, indicative of a random coil with partial helical character. The addition of 50 µM of 34RH did not change the conformation of MaxRH. The CD spectrum of MaxRH is consistent with it being a monomer at 1 µM, presumably due to the introduced Myc residues reducing the homodimer stability of the parental P22 Max sequence [54,56]. In order to isolate the fluorescence of the tyrosine in the binding site from the signal of the two C-terminal tyrosine residues in MaxRH, we mutated these residues to phenylalanine to generate MaxRH-Y115F/Y123F. We observed that MaxRH-Y115F/Y123F fluorescence was quenched with 50 µM 34RH (Figure 4D) on par with the quenching seen with Myc_353–437_ and Myc_402–412_ confirming that the Tyr in the binding site of MaxRH is the residue quenched upon binding and that the quenching is similar to that seen in the native Myc context. From the titration of MaxRH-Y115F/Y123F with 34RH, we obtained a K_D_ of 14.9 ± 1.9 µM (Figure 4E). We also obtained the CD spectra of MaxRH-Y115F/Y123F in the presence and absence of 50 µM 34RH and confirmed that the protein remains disordered even in the presence of the small molecule (Figure 4F).

We next tested P22 Max to verify that Max does not bind to 34RH. We observed that the tyrosine fluorescence of P22 Max does not exhibit titratable quenching with 34RH (Figure 4G,H). The CD of P22 Max with and without 34RH indicated that 34RH does not alter the CD of P22 Max. The spectra do, however, exhibit a substantially greater helical character of P22 Max, indicative of homodimer formation (Figure 4I) [57]. In a homodimer, Tyr_115_ and Tyr_123_ would still be expected to be accessible to 34RH; however, Tyr_70_ and adjacent residues would likely be occluded by the dimer structure.

**Figure 5:**
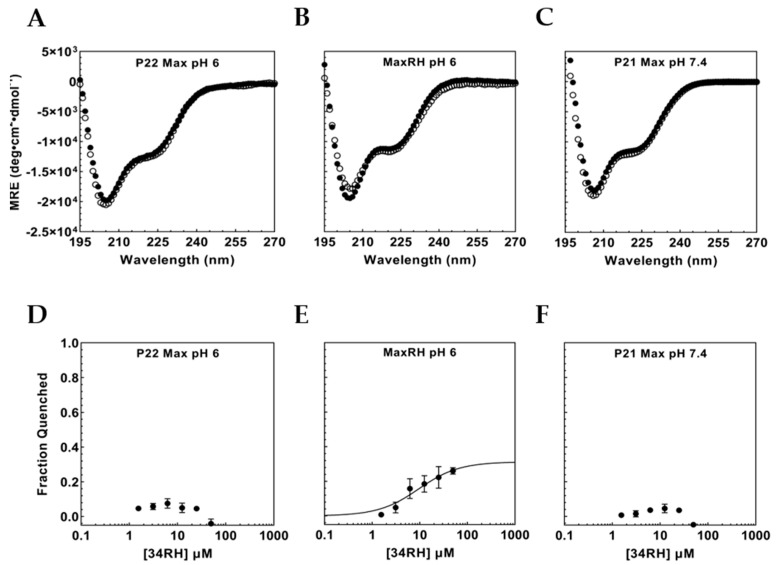
(**A**) CD of 1 µM P22 Max at pH 6 with (black circles) and without (white circles) 50 µM 34RH. (**B**) CD of 1 µM MaxRH at pH 6 with (black circles) and without (white circles) 50 µM 34RH. (**C**) CD of 4 µM P21 Max at pH 7.4 with (black circles) and without (white circles) 50 µM 34RH. (**D**) Fraction quenched titration curve for 1 µM P22 Max with 34RH at pH 6. (**E**) Fraction quenched titration curve for 1 µM MaxRH with 34RH at pH 6 fitted to a Langmuir binding isotherm, K_D_ = 9.1 ± 3.9 µM (**F**) Fraction quenched titration curve for 1 µM P21 Max with 34RH at pH 7.4. Error bars represent the standard error of three independent trials.

To control for binding interactions of 34RH with the P22 Max sequence in a monomeric state, titrations were conducted at pH 6. The lower pH disfavors dimer formation leading to monomeric P22 Max [58]. At pH 6, CD results with P22 Max indicated a substantial loss in helical character, with a spectrum similar to Myc_353–437_ and MaxRH, and consistent with the monomeric form of P22 Max (Figure 5A). Fluorescence experiments with 34RH and P22 Max were performed at pH 6 (Figure 5D) and again showed no titratable quenching of P22 Max fluorescence. To confirm binding still occurs under these conditions, MaxRH fluorescence quenching and CD were measured at pH 6 (Figure 5B,E). At pH 6, MaxRH still bound to 34RH and actually improved in affinity with a dissociation constant of 9.1 ± 3.9 µM while remaining disordered as observed via CD. As a further control, we also tested for binding to the 151 residue P21 isoform of Max. The nine-residue difference at the N-terminus (prior to the bHLHZip) between the two Max isoforms is associated with a weaker homodimerization constant for P21 Max [59]. The CD spectrum of P21 Max at pH 7.4 was consistent with a monomeric state with no indication of perturbation in the presence of 34RH (Figure 5C). The tyrosine fluorescence of P21 Max versus 34RH concentration was similar to results with P22 Max showing no titratable quenching (Figure 5F). These results indicated that the native Max sequence does not interact with 34RH in regions around its tyrosine residues and demonstrated that the 34RH binding function was ported into the Max context by introduction of a minimal binding sequence.

### 3.4. Flanking Residues Modulate 34RH Binding

**Figure 6:**
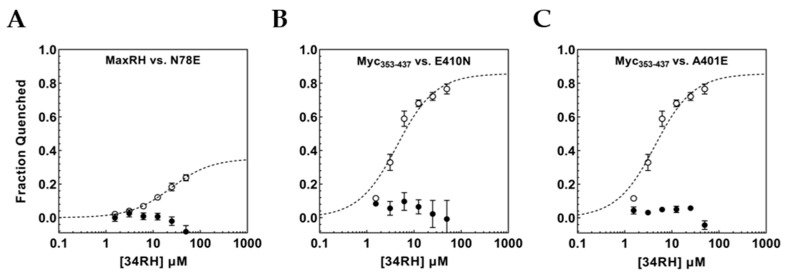
(**A**) MaxRH-N78E fluorescence quenching titration curve (black circles) overlaid with MaxRH curve (white circles). (**B**) Myc_353–437_ E410N fluorescence quenching titration curve (black circles) overlaid with Myc_353–437_ curve (white circles). (**C**) Myc_353–437_ A401E fluorescence quenching titration curve (black circles) overlaid with Myc_353–437_ curve (white circles). Error bars represent the standard error of three independent trials.

At pH 7.4, the K_D_ obtained for MaxRH:34RH binding was notably higher than the value observed with Myc_353–437_. The Myc_353–437_ and MaxRH sequences differ in the flanking residues directly adjacent to the binding site. Using point mutations, we wanted to examine the impact of flanking residues on the minimal binding site in Myc_353–437_ and MaxRH. At the C-terminus, MaxRH has an asparagine directly adjacent to the binding site while Myc_353–437_ has a glutamic acid. We wanted to test if mutating N_78_ in MaxRH to a glutamic acid would improve binding. The MaxRH-N78E mutant extended the MaxRH:Myc_353–437_ identity by one residue flanking the binding site (-Y_70_ILSVQAEE_78_-). Surprisingly, the mutation caused a complete loss of observable binding with no titratable tyrosine quenching (Figure 6A). Since a flanking Asn permitted 34RH binding in MaxRH while Glu eliminated it, the reciprocal mutation was tested in Myc_353–437_. The construct Myc_353–437_ E410N was tested for binding to 34RH. Here again, a relatively conservative change in the residue flanking the binding site eliminated binding to 34RH (Figure 6B). The identity of the C-terminal flanking residue had opposing effects in the Myc and MaxRH contexts. These sequences diverge on the C-terminal side of the binding site showing little sequence identity (Figure 3). At the N-terminal side, however, five out of seven residues adjacent to the binding site are identical between Myc and MaxRH. Directly flanking the Tyr of the binding site, MaxRH has a glutamic acid while Myc has an alanine. We constructed Myc_353–437_ A401E to determine if the same flanking residue would be permissive of binding in both protein contexts at the N-terminal side. The titration of Myc_353–437_ A401E with 34RH caused no detectable binding (Figure 6C). Here again, we observed a flanking residue that was permissive of binding in one context but eliminated binding in the other.

## 4. Discussion

Short stretches of disordered regions have been shown to bind to small molecules with at least micromolar affinity [40]. SLiMs also engage in molecular recognition via short, localized sequences, are typically present in disordered regions, and typically bind to their partner proteins with micromolar affinity. An inherent characteristic of SLiMs is their modularity and resulting portability [27]. Based on analogous aspects between SLiMs and small-molecule binding sites in disordered proteins, we believed that small-molecule binding sites could also show portability and allow their binding function to move between protein contexts as the short binding sequence is moved.

Using the small molecule 34RH, we demonstrated that the binding observed in the context of Myc_353–437_ is maintained with only a moderate (3-fold) change in affinity for the binding site in the isolated peptide sequence Myc_402–412_, similar to what was previously observed for the 1RH compound [49]. NMR data from Panova and coworkers have indicated that Myc_353–437_ is expected to be compact with paramagnetic relaxation enhancement (PRE) data showing contacts between residue 400–412 and 360–380, along with some predicted helical character (~20%) in the 400–412 region [51]. In contrast, the Myc_402–412_ peptide is a random coil that lacks a surrounding protein context and so is devoid of additional contacts with the protein sequence. Despite these differences, the affinity for 34RH in the two contexts differs by less than 0.7 kcal mol^−1^, indicating substantial modularity to the small molecule binding sequence.

By mutating six residues in Max to produce MaxRH, we transferred a small molecule binding site into a new protein context and could observe binding. The affinity of 34RH was about 6-fold weaker than in the Myc context (2-fold weaker relative to the peptide). At pH 6, the binding of 34RH to MaxRH improved 2.5-fold to a K_D_ of 9.1 μM. Kizilsavas and coworkers had studied monomeric Max via NMR under similar conditions (pH 5.5) and found the sequence to be disordered but highly compact [60]. These results demonstrate that small molecule binding sites can exhibit portability between disordered protein contexts. Furthermore, the binding can be robust to variations in the conformational propensity and surrounding protein environment with only several-fold variation in affinity when the binding site is in a very compact disordered domain (Max), a partially ordered domain with tertiary contacts (Myc), or in a short peptide sequence. The protein context can tune the binding, but in the absence of a disorder to order transition [28], it does not appear to be a major factor or even a necessary component for binding [40].

The eight-residue sequence from Myc (YILSVQAE) was found to be sufficient to transfer binding function when placed in the context of the Max sequence; however, in both Myc and MaxRH, binding was very sensitive to the identity of the immediately flanking residue at both ends of the sequence. In MaxRH, mutating the C-terminal flanking Asn to Glu eliminated detectable binding while in the Myc context we observed a reciprocal effect. Mutating the flanking Glu to Asn eliminated binding to Myc. A residue that was permissive of binding in one context was prohibitive in the other. At the N-terminal end of the binding sequence in Myc_353–437_, we observed a similar effect; mutating the native Ala to a Glu, which is present in the equivalent position in MaxRH, eliminated binding. Truncations can define a minimal necessary sequence for small-molecule binding to a peptide but that may not be sufficient for binding in a given protein domain context. Flanking residues have been shown previously to influence the binding of adjacent disordered sequences [61,62]. Despite remaining disordered in the complex, small-molecule binding affinity can also be strongly influenced by flanking residues.

Here, we show that a disordered small-molecule binding site can be ported between disordered protein contexts and retain its binding function. This finding supports the idea that if we are able to identify minimal sequences that can bind small molecules, then these sequences are likely to retain their binding function when in the context of various disordered domains. However, we also find that residues flanking the set of necessary binding residues can influence binding, with the same flanking residue being either permissive or prohibitive of binding depending on the broader protein context. While the influence of flanking residues increases the complexity of identifying the small-molecule binding sites, it also increases the specificity of the binding site by increasing the sequence requirements needed to achieve binding

## Data Availability

Not applicable.

## Supplementary Materials

The following supporting information can be downloaded at: https://www.mdpi.com/article/10.3390/biom12121887/s1, Figure S1: Myc, MaxRH, P22 Max, and P21 Max constructs aligned using CLUSTAL O (1.2.4) multiple sequence alignment [63]; Figure S2: UV/Vis characterization of 34RH [45]; Figure S3: Dynamic Light Scattering of 34RH in 1xPBS (buffer) at pH 7.4; Figure S4: Myc_353–437_ and 34RH interaction monitored via tyrosine fluorescence quenching; Figure S5: Equimolar Myc_353–437_ and 34RH interaction monitored via tyrosine fluorescence quenching [46,49]; Figure S6: NMR characterization of 34RH.
