# Supplementary material for: Portability of a Small-Molecule Binding Site between Disordered Proteins
Source: Biomolecules. 2022 Dec 16;12(12):1887. doi: 10.3390/biom12121887 (PMC9775153; doi:10.3390/biom12121887)
Supplement: Supplementary file 1 [file biomolecules-12-01887-s001.zip › biomolecules-1979698-supplementary.pdf]

Supplementary Material

Portability of a Small Molecule Binding Site Between Disordered Proteins

Rajesh Jaiprashad, Sachith Roch De Silva, Lisette M. Fred Lucena, Ella Meyer, Steven J. Metallo

Table of Contents

Fig S1. Myc, MaxRH, MaxRH-Y115F/Y123F, P22 Max, and P21 Max constructs aligned using CLUSTAL O (1.2.4) multiple sequence alignment

Fig S2. UV/Vis characterization of 34RH

Fig S3. Dynamic light scattering of 34RH in 1xPBS (buffer) at pH 7.4

Fig S4. $Myc_{353-437}$ and 34RH interaction monitored via tyrosine fluorescence quenching and free fluorescence spectra of $Myc_{353-437}$ and 34RH

Fig S5. Equimolar $Myc_{353-437}$ and 34RH interaction monitored via tyrosine fluorescence quenching

Fig S6. NMR characterization of 34RH

```
Myc         -------------------------gsNVKRRTHNVLERQRRNELKRSFFALRDQIPELE   385
MaxRH       -----gMSDNDDIEVESDEEQPRFQSAADKRAHHNALERKRRDHIKDSFHSLRDSVPSLQ   54
MaxRH*      -----gMSDNDDIEVESDEEQPRFQSAADKRAHHNALERKRRDHIKDSFHSLRDSVPSLQ   54
P22         gidpftMSDNDDIEVESDEEQPRFQSAADKRAHHNALERKRRDHIKDSFHSLRDSVPSLQ   54
P21         gidpftMSDNDDIEVESD---------ADKRAHHNALERKRRDHIKDSFHSLRDSVPSLQ   45
                                         **  **.***:**:.:* **.:***.:*.*:

Myc         NNEKAPKVVILKKATAYILSVQAEEQKLISEEDLLRKRREQLKHKLEQL---RNS-----   437
MaxRH       G-EKASRAQILDKATEYILSVQAENHTHQQDIDDLKRQNALLEQQVRALEKARSSAQLQT   113
MaxRH*      G-EKASRAQILDKATEYILSVQAENHTHQQDIDDLKRQNALLEQQVRALEKARSSAQLQT   113
P22         G-EKASRAQILDKATEYIQYMRRKNHTHQQDIDDLKRQNALLEQQVRALEKARSSAQLQT   113
P21         G-EKASRAQILDKATEYIQYMRRKNHTHQQDIDDLKRQNALLEQQVRALEKARSSAQLQT   104
            . *** :. **.*** **  :: :::.  .: * *:::.  *::::. *   *.*

Myc         -----------------------------------------------                ---
MaxRH       NYPSSDNSLYTNAKGSTISAFDGGSDSSSESEPEEPQSRKKLRMEAS                160
MaxRH*      NFPSSDNSLFTNAKGSTISAFDGGSDSSSESEPEEPQSRKKLRMEAS                160
P22         NYPSSDNSLYTNAKGSTISAFDGGSDSSSESEPEEPQSRKKLRMEAS                160
P21         NYPSSDNSLYTNAKGSTISAFDGGSDSSSESEPEEPQSRKKLRMEAS                151
```

Fig S1. Myc, MaxRH, MaxRH-Y115F/Y123F (denoted as MaxRH* in figure), P22 Max, and P21 Max constructs aligned using CLUSTAL O (1.2.4) multiple sequence alignment. Lowercase letters denote non-native residues remaining after TEV cleavage [63].

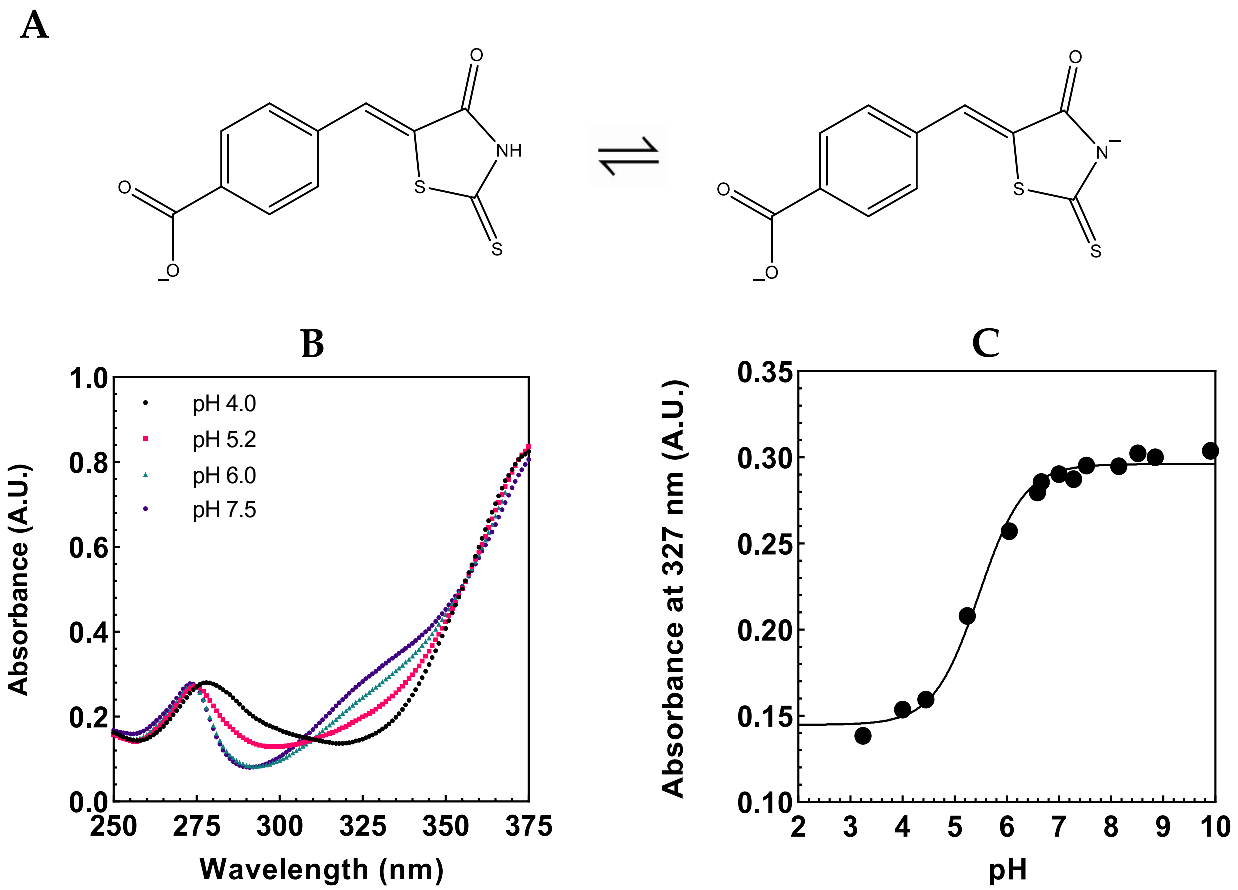

Fig S2. UV/Vis characterization of 34RH. (**A**) pH dependent deprotonation of 34RH. (**B**) UV/Vis absorbance of 10 μM 34RH in 1xPBS, 5% DMSO, 25 °C, at pH 4.0 (black), pH 5.2 (dark pink), pH 6.0 (teal), and pH 7.5 (purple). (**C)** 34RH absorbance at 327 nm at various pH values showing a pH dependent titration fit to the Henderson-Hasselbalch equation [45].

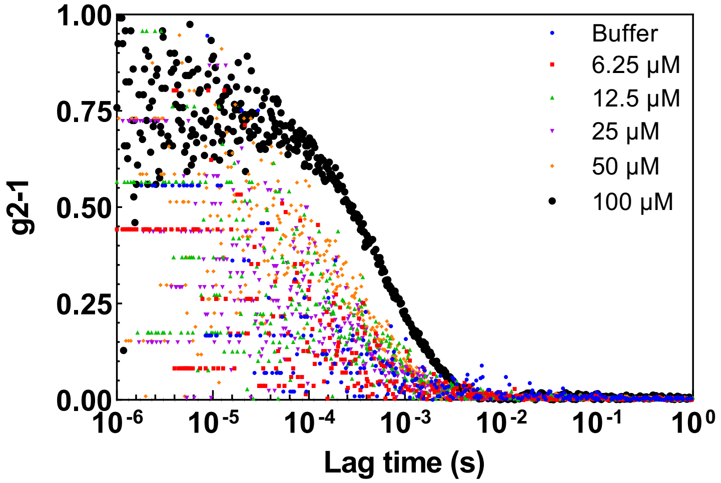

Fig S3. Dynamic Light Scattering of 34RH in 1xPBS and 5% DMSO (buffer) at pH 7.4. Concentrations of 34RH are shown in the upper right corner. Concentrations below 50 µM show no correlation function indicating that there are none to minimal nano-sized particles in the samples. At 100 µM, a correlation was observed indicating that nano-sized particles may exist in solution. All experiments were carried out at 50 µM and below to avoid any potential particle interference in the data.

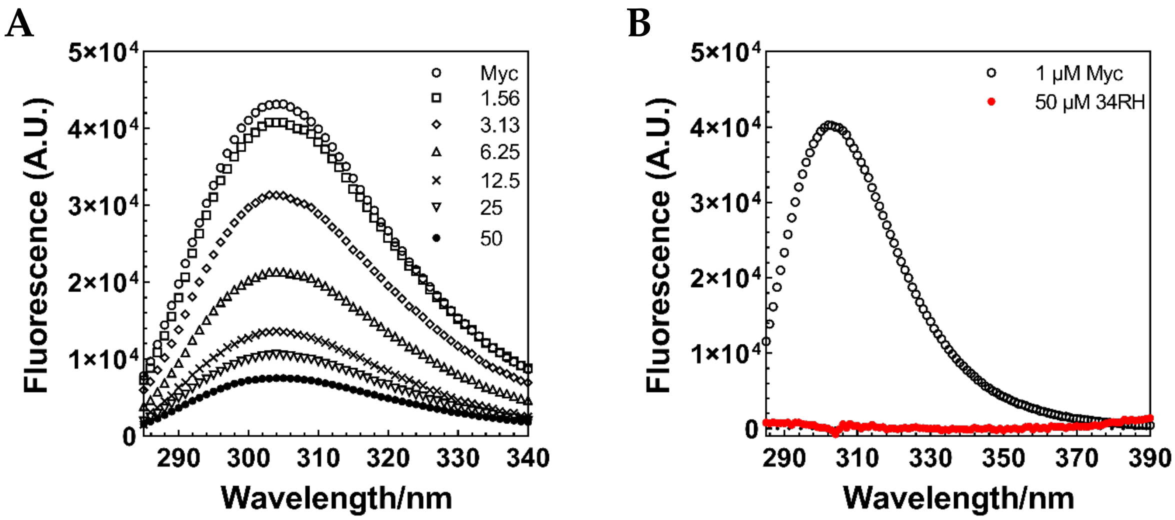

Fig S4. $Myc_{353-437}$ and 34RH interaction monitored via tyrosine fluorescence quenching and free fluorescence spectra of $Myc_{353-437}$ and 34RH. (A) Inner filter corrected fluorescence emission spectra of 1 µM $Myc_{353-437}$ (open circles) and 1 µM $Myc_{353-437}$ with various concentrations of 34RH in 1xPBS, 5% DMSO at 25 °C, pH 7.4. Concentrations of 34RH (in micromolar) are shown in the upper right corner. (B) Comparison of inner filter corrected emission spectra of 1 µM $Myc_{353-437}$ (open circles) and 50 µM 34RH without Myc (closed circles) demonstrating no fluorescence contribution from 34RH in this wavelength range.

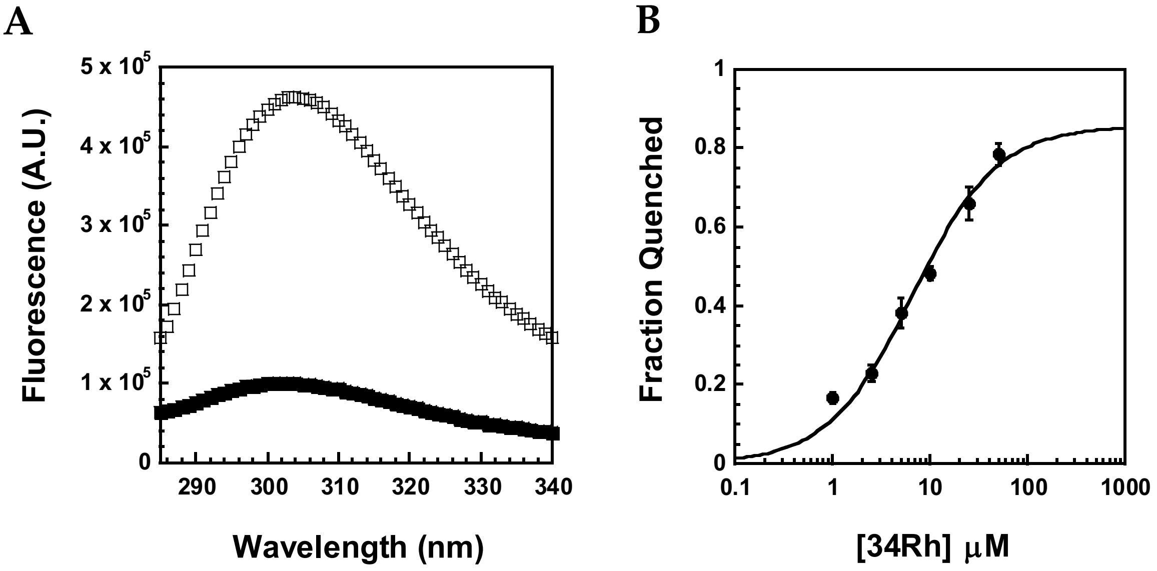

Fig S5. Equimolar $Myc_{353\text{-}437}$ and 34RH interaction monitored via tyrosine fluorescence quenching. **(A)** 50 μM $Myc_{353\text{-}437}$ in the presence (filled black squares) and absence (open black squares) equimolar 34RH in 1x PBS at 25 °C, pH 7.4. (**B)** Equilibrium quenching of an equimolar titration of Myc353-437 and 34RH fit to a 1:1 binding model, K_D of 5.9 ± 0.8 μM. Error bars represent the standard error of three independent trials. Representative fluorescence signals were inner filter corrected to account for any fluorescence suppression due the absorbance of protein and 34RH [46]. The quenching data was fitted to a binding model shown below described in Dobrev et al.

$$\text{Fraction Quenched} = Q_{max} \times \frac{2+\frac{K_D}{[C]_{tot}} - \sqrt{\left(-2-\frac{K_D}{[C]_{tot}}\right)^2 - 4}}{2}$$

Here, Q_{max} is the fraction quenched for the formation of the complete complex between $Myc_{353\text{-}437}$ and 34RH. $[C]_{tot}$ is the total concentration of $Myc_{353\text{-}437}$ or 34RH and K_D the dissociation constant. [46,49]

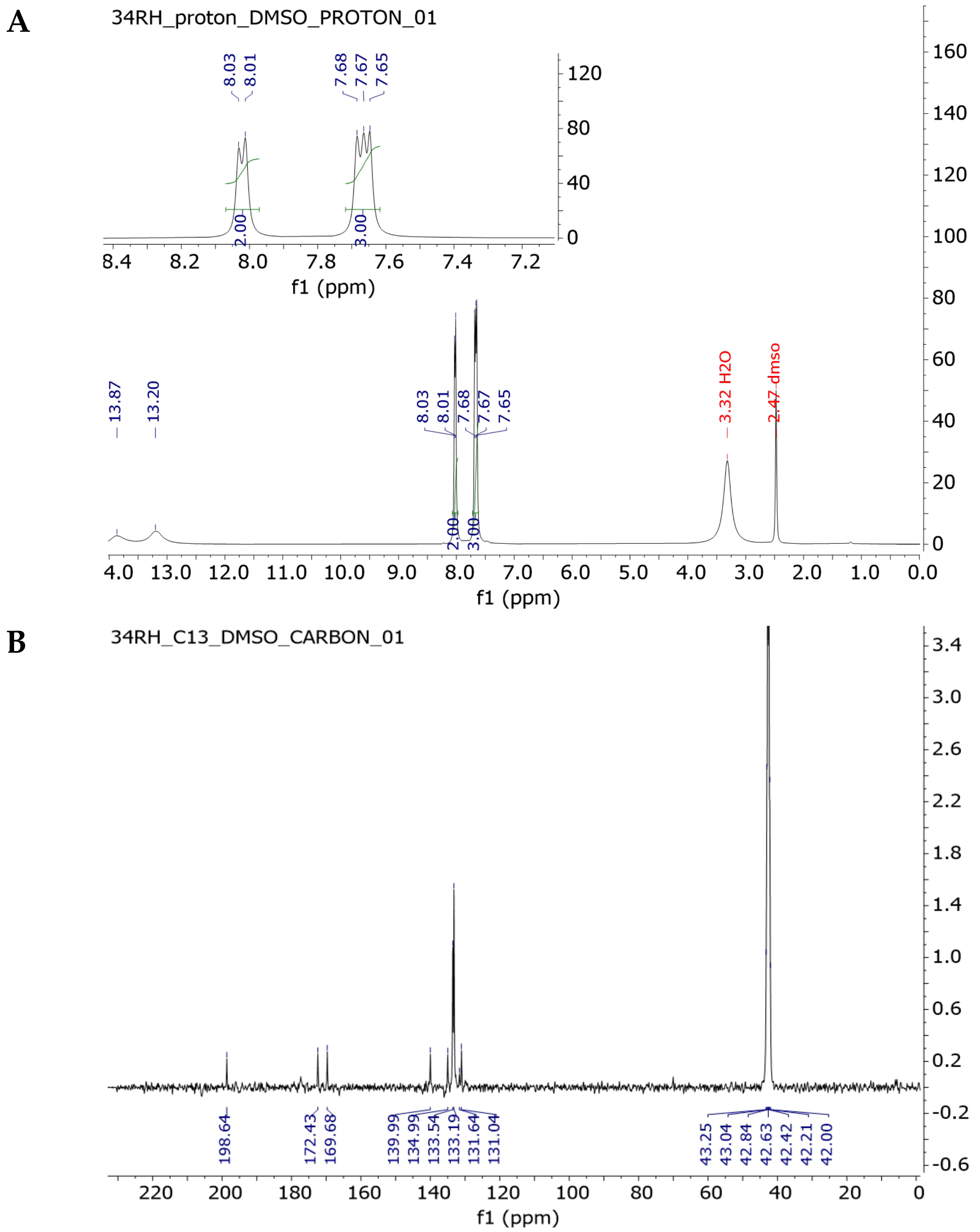

Fig S6. NMR characterization of 34RH **A)** 1H NMR of 34RH in DMSO-d6. **B)** 13C NMR of 34RH in DMSO-d6.

References

45. Luiz, F.C.L.; Louro, S.R.W. Acid-base equilibrium of drugs in time-resolved fluorescence measurements: Theoretical aspects and expressions for apparent pK(a) shifts. *J Photoch Photobio A* **2011**, 222, 10-15, doi:10.1016/j.jphotochem.2011.03.006.
46. Dobrev, V. S.; Fred, L. M.; Gerhart, K. P.; Metallo, S. J. *Characterization of the Binding of Small Molecules to Intrinsically Disordered Proteins*, 1st ed.; Elsevier Inc., **2018**; Vol. 611. https://doi.org/10.1016/bs.mie.2018.09.033.
49. Follis, A. V.; Hammoudeh, D. I.; Wang, H.; Prochownik, E. V.; Metallo, S. J. Structural Rationale for the Coupled Binding and Unfolding of the C-Myc Oncoprotein by Small Molecules. *Chem. Biol.* **2008**, *15* (11), 1149–1155. https://doi.org/10.1016/j.chembiol.2008.09.011.
63. Sievers, F.; Wilm, A.; Dineen, D.; Gibson, T. J.; Karplus, K.; Li, W.; Lopez, R.; McWilliam, H.; Remmert, M.; Söding, J.; et al. Fast, Scalable Generation of High-Quality Protein Multiple Sequence Alignments Using Clustal Omega. Mol. Syst. Biol. 2011, 7 (539). https://doi.org/10.1038/msb.2011.75.
